# p–i–n Perovskite Solar Cells on Steel Substrates

**DOI:** 10.1021/acsaem.2c00291

**Published:** 2022-06-14

**Authors:** Benjamin T. Feleki, Ricardo K. M. Bouwer, Valerio Zardetto, Martijn M. Wienk, René A. J. Janssen

**Affiliations:** † Molecular Materials and Nanosystems and Institute for Complex Molecular Systems, Eindhoven University of Technology, P.O. Box 513, 5600 MB Eindhoven, The Netherlands; ‡ Tata Steel, Research and Development, Surface Engineering−Coating Development, IJmuiden 1970 CA, The Netherlands; § TNO, Partner in Solliance, High Tech Campus 21, 5656 AE Eindhoven, The Netherlands; ∥ Dutch Institute for Fundamental Energy Research, De Zaale 20, 5612 AJ Eindhoven, The Netherlands

**Keywords:** metal-halide perovskites, optical modeling, solar cells, steel substrates, substrate-configuration solar cells, building-integrated photovoltaics, triple-cation perovskite.

## Abstract

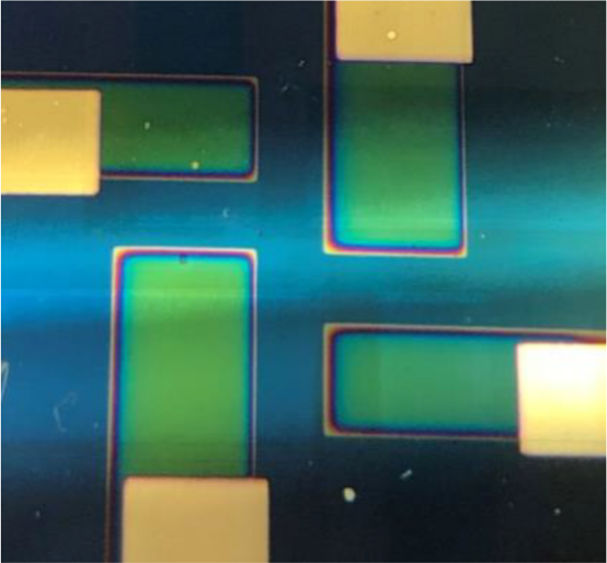

An efficient substrate-configuration p–i–n metal-halide perovskite solar cell (PSC) is fabricated on a polymer-coated steel substrate. The optimized cell employs a Ti bottom electrode coated with a thin indium tin oxide (ITO) interlayer covered with a self-assembled [2-(9*H*-carbazol-9-yl)ethyl]phosphonic acid monolayer as a hole-selective contact. A triple-cation perovskite is used as the absorber layer. Thermally evaporated C_60_ and atomic layer deposited SnO_2_ layers serve to create an electron-selective contact. The cells use an ITO top electrode with an antireflective MgF_2_ coating. The optimized cell fabricated on a polymer-coated steel substrate reaches a power conversion efficiency of 16.5%, which approaches the 18.4% efficiency of a p–i–n reference superstrate-configuration cell that uses a similar stack design. Optical simulations suggest that the remaining optical losses are due to the absorption of light by the ITO top electrode, the C_60_ layer, the Ti bottom electrode, and reflection from the MgF_2_ coating in almost equal amounts. The major loss is, however, in the fill factor as a result of an increased sheet resistance of the top ITO electrode.

## 1 Introduction

The large, often unused opaque areas of rooftops and façades of warehouses, logistic centers, and production halls can be used to provide sustainable electricity production when covered with photovoltaic modules. These industrial buildings often employ coated steel as the building skin. Hence, it is of interest to consider steel as a substrate for fabricating photovoltaic cells. Because of their low cost, light weight, high efficiency, and compatibility with a variety of substrates,^1^ metal-halide perovskite solar cells (PSCs) can possibly provide a technology for building-integrated photovoltaics when fabricated directly on steel.^2^ When PSCs are fabricated on a metal foil or rigid substrate, a so-called substrate configuration is required in which the cell is illuminated via a transparent top electrode, similar to semi-transparent solar cells.^3^ Achieving high power conversion efficiencies (PCEs) has been challenging for substrate-configuration PSCs deposited on metal substrates.^4−20^ Substrate-configurations have also been designed for perovskite photovoltaic metal fibers.^21−23^ The highest reported efficiencies for PSCs on metal substrates range from 14.7 to 15.2% for substrate-configuration PSCs on metal substrates^12,13,19^ and are less than for superstrate-configuration architectures, where the PSC is built on and illuminated through a transparent glass substrate. Superstrate-configuration PSCs recently reached a record PCE of 25.7%.^24^ This large difference in PCE not necessarily reflects intrinsic limitations but is also caused by the fact that much less effort has been given to substrate-configurations compared to superstrate-configurations.

Most PSCs fabricated on metal foils use a n–i–p cell architecture and reports for p–i–n stacks are scarce.^11,15^ The highest efficiency reported for such substrate p–i–n PSCs is 12.8%, employing a Cu foil bottom electrode and a Ag nanowire transparent top electrode.^11^ Interestingly, the p–i–n substrate-configuration is now commonly in use for wide-bandgap PSCs as part of two-terminal monolithic tandem solar cells with a crystalline Si bottom cell.^25−28^ Moreover, for p–i–n PSCs, several charge-selective contacts are known that provide low resistive losses and excellent energetic alignment with the perovskite active layer.^29−31^ A recent study demonstrated self-assembled monolayers (SAMs) consisting of [2-(9*H*-carbazol-9-yl)ethyl]phosphonic acid (2PACz) on indium tin oxide (ITO) as the hole-selective contact in combination with a thermally evaporated C_60_ layer as the electron-selective contact.^31^

Herein, we demonstrate an efficient substrate-configuration p–i–n PSC on steel coated with a polyamide-imide (PAI) planarization layer. We chose to use a Ni-plated steel substrate because it is relatively smooth and presents moderate macroscopic roughness^20^ and excellent chemical resistance. Ni-plated steel substrates are commonly used in batteries.^32^ The planarization layer serves as an insulating layer. An opaque titanium electrode covered with a thin sputtered ITO layer to enable binding of the phosphonic acid anchoring groups of the 2PACz monolayer serves as a hole-collecting electrode. For electron collection, we use a C_60_ layer covered with a SnO_2_ buffer layer that prevents damage to the underlying stack during sputter deposition of the transparent ITO top electrode.^33,34^ Optimized devices on steel substrates reach an efficiency of 16.5%. While the cell outperforms the present record performance for p–i–n and n–i–p cells on opaque substrates, the efficiency is still lower than the 18.4% efficiency obtained for the corresponding superstrate p–i–n solar cell. Optical modeling is used to analyze parasitic optical losses.

## 2 Experimental Section

### 2.1 Materials and Solution Preparation

All materials and reagents were purchased from commercial sources. Solutions were stirred at 60 °C overnight before the spin coating, unless stated otherwise. 2PACz (TCI Chemicals, >98.0%) was dissolved in absolute ethanol (Biosolve, AR grade) at a concentration of 0.33 mg mL^–1^. The solution was sonicated for 30 min before deposition. For the active layer, a triple-cation Cs_0.05_(MA_0.17_FA_0.83_)_0.95_Pb(I_0.83_Br_0.17_)_3_ perovskite was used as described by Saliba et al.^36^ PbI_2_ (576 mg) (TCI Chemicals, 99.99% trace metal basis) and PbBr_2_ (550.5 mg) (TCI Chemicals, 99.99% trace metal basis) were dissolved separately in a mixture of *N*,*N*-dimethylformamide (DMF, 0.8 mL) and dimethyl sulfoxide (DMSO, 0.2 mL). Then, 0.936 mL of the PbI_2_ solution was added to FAI (200 mg) (Greatcell Solar), and 0.702 mL of the PbBr_2_ solution was added to MABr (99.7 mg) (Greatcell Solar). Finally, 0.833 mL of the PbI_2_-FAI solution, 0.167 mL of the PbBr_2_-MABr, and 50 μL of CsI (Sigma Aldrich, 99.999%) of a stock solution of 389.7 mg mL^–1^ in DMSO (Sigma Aldrich, anhydrous 99.9%) were mixed. As the electron-transport layer (ETL), a combination of C_60_ (SES Research, 99.95%) and a spatial atomic layer deposition (ALD) SnO_2_ layer or bathocuproine (BCP) (Lumtec, 99%) was used. The ITO sputter target (purity 99.95%) for top electrodes was purchased from Angstrom Engineering. As the antireflective coating, MgF_2_ (Alfa Aesar, 99.995%) was used.

### 2.2 Device Fabrication

All thermally evaporated films were deposited under high-vacuum conditions at ∼5 × 10^–7^ mbar. Pre-patterned ITO (180 nm) glass substrates (Naranjo Substrates) were cleaned by sonication in acetone (15 min), scrubbing and sonication in sodium dodecyl sulfate solution (Acros, 99%) in water (10 min), rinsing in deionized water, and sonication in 2-propanol (15 min). Prior to device preparation, the glass substrates were blow-dried with nitrogen and activated by UV-ozone treatment (30 min). Ni-plated battery steel (HILUMIN, Tata Steel) substrates were cleaned in 2-propanol and blow-dried with N_2_. On the steel substrate, a wire bar-coated PAI (Torlon Al-10, Solvay) planarization layer was used. The PAI film was cured in air at 265 °C for 15 min and cut to 3 × 3 cm^2^ samples for further use. Prior to the bottom electrode deposition, the samples were sonicated in 2-propanol for 15 min and blow-dried with N_2_. The solar cell fabrication on the planarized steel substrates was identical to the fabrication of glass. For substrate-configuration PSCs, a 200 nm patterned Ti bottom electrode was deposited (2 Å s^–1^) onto the glass/ITO and PAI-coated steel substrates via electron-beam deposition. A 10 nm patterned ITO interlayer was deposited (∼0.3 Å s^–1^) onto the Ti bottom electrode via magnetron sputtering under an Ar/O_2_ flow.

The 2PACz solution was statically spin-coated onto the ITO interlayer at 3000 rpm (with a 20,000 rpm s^–1^ acceleration) for 30 s and heat-treated at 100 °C for 10 min.

The Cs_0.05_(MA_0.17_FA_0.83_)_0.95_Pb(I_0.83_Br_0.17_)_3_ perovskite film (∼520 nm thick) was processed using a ramped spin-coating deposition. The perovskite precursor solution was deposited statically onto the hole-transport layer (HTL) at 4000 rpm (800 rpm s^–1^) for 35 s. 10 s prior to the end of the spin-coating program, 300 μL of anhydrous ethyl acetate (Sigma Aldrich, 99.8%) was deposited. The perovskite film was then annealed in a glovebox at 100 °C for 60 min and cooled to room temperature.

A 20 nm C_60_ layer was deposited (2 Å s^–1^) as the ETL on the perovskite films via thermal evaporation. For substrate-configuration cells, a 45 nm thick SnO_2_ was grown on the C_60_ layer by spatial ALD as described elsewhere.^35^ Tetrakis(dimethylamino)tin(IV) was the Sn source and H_2_O was used as the coreactant. Both vessels were kept at room temperature while flowing 500 sccm of Ar through them. Processing was done at 100 °C with a nominal growth of 0.125 nm/cycle, determined on a silicon wafer. After SnO_2_ deposition, the samples were transferred into a N_2_-filled glovebox for ITO sputtering. The ITO top electrode (180 nm) was deposited (∼0.3 Å s^–1^) using radio frequency sputtering under Ar/O_2_ flow. The antireflective MgF_2_ coating (90 nm) was deposited (2 Å s^–1^) via thermal evaporation. The superstrate-configuration cell of the device was finalized by a BCP (8 nm) layer and a 100 nm Ag top electrode which were deposited (2 Å s^–1^ both) via thermal evaporation. The active area (0.09 cm^2^ or 0.16 cm^2^) of the cells was determined by the overlap of the ITO or Ti bottom electrode and the transparent ITO or Ag top electrode.

### 2.3 Device Characterization

All samples were stored and measured in a nitrogen-filled glovebox without any further exposure to air or any preconditioning, unless stated otherwise. The current density–voltage (*J–V*) characteristics were measured using a Keithley 2400 source meter. During the *J–V* measurements, light from a tungsten-halogen lamp was filtered using a Schott GG385 UV filter and a Hoya LB120 daylight filter to mimic the AM1.5G spectrum (100 mW cm^–2^). For ITO/MgF_2_ side and glass ITO side illuminated solar cells, a black shadow mask with an aperture area of 0.0676 or 0.1296 cm^2^ was employed to define the illuminated cell area. During the fast *J–V* sweep measurements, the source meter swept the voltage either from +1.5 to −0.5 V (reverse scan) or from −0.5 to +1.5 V (forward scan) at a scan rate of 0.25 V s^–1^. Light soaking preconditioning of the solar cells was performed by exposing the cell area to continuous illumination of simulated AM1.5G (100 mW cm^–2^) light for a given time, followed by a fast sweep measurement. For the stabilized *J–V* measurement (slow sweep measurements), the open-circuit voltage (*V*_oc_) of the solar cell was first tracked for 5 min under constant illumination. Then, a reverse sweep from *V*_oc_ +0.04 V to −0.04 V was performed with a step size of 0.04 V in which the current density was measured at each voltage step after a stabilization time of 5 s.

External quantum efficiency (EQE) measurements were performed in a N_2_ atmosphere. The probe light was generated by a 50 W tungsten-halogen lamp (Philips Focusline), which was modulated with a mechanical chopper (Stanford Research, SR 540) before passing through a monochromator (Oriel, Cornerstone 130). The spectral response of the device was recorded as a voltage from a pre-amplifier (Stanford Research, SR 570) using a lock-in amplifier (Stanford Research, SR 830) and was calibrated by a reference silicon cell. To accurately determine the short-circuit current density (*J*_sc,EQE_), a green LED (530 nm, Thorlabs M530L3, driven by a DC4104 driver) was utilized as a light bias during the EQE measurement to provide the solar cell with approximately 1 sun equivalent illumination intensity.

### 2.4 Optical Simulations

Optical simulations were performed using the transfer-matrix method with Setfos 5.0 (Fluxim AG). The wavelength-dependent refractive index (*n*) and extinction coefficient (*k*) used for the different materials are shown in the Supporting Information.

## 3 Results and Discussion

**Figure 1:**
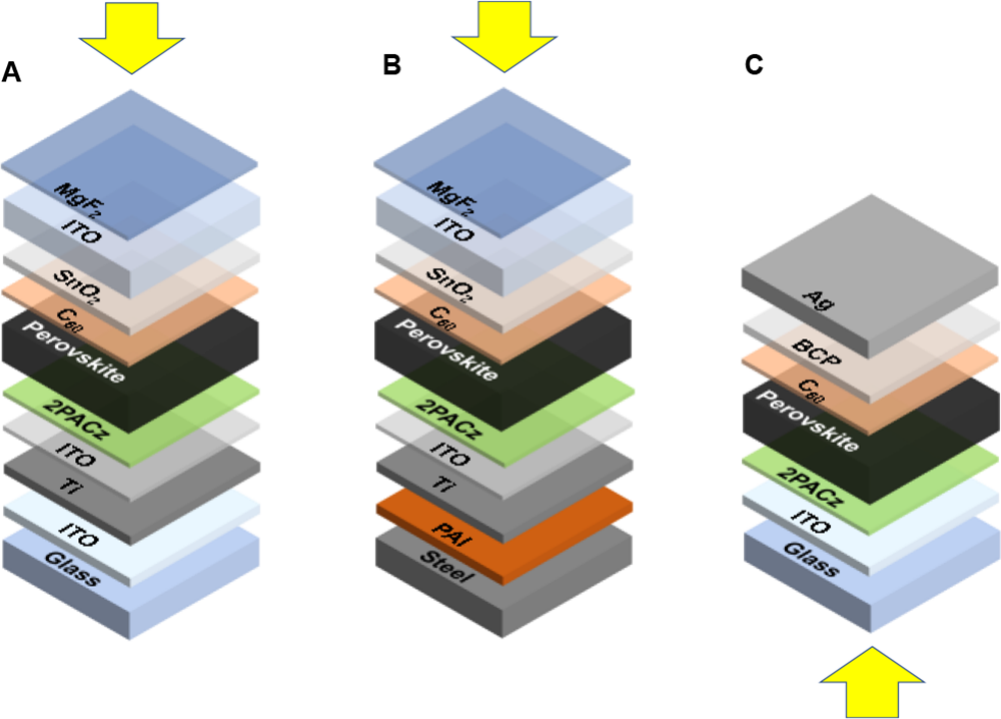
Substrate (A and B) and superstrate (C) p–i–n solar cells on glass (A and C) and steel (B). The arrows indicate the illumination direction. Layer thicknesses: glass (750 μm), ITO bottom (170 nm), Ti (200 nm), ITO interlayer (10 nm), 2PACz (monolayer), perovskite (520 nm), C_60_ (20 nm), SnO_2_ (45 nm), top ITO (80 nm), MgF_2_ (90 nm), steel (250 μm), PAI (5 μm), BCP (8 nm), and Ag (100 nm).

To investigate the effect of the substrate on the performance, we built cells on glass and steel substrates (Figure 1, cells A and B). Ni-plated battery steel (250 μm) planarized by an insulating PAI (5 μm) layer was used as the steel substrate. The maximum profile peak height above the mean line (*R*_p_), determined with surface profilometry on a 2 × 2 mm^2^ surface area, of the PAI-coated steel substrates is 510 nm. Locally, the surface is smoother and *R*_p_ is 10 nm when measured with atomic force microscopy on a 5 × 5 μm^2^ area. Electron-beam evaporation was used to deposit a Ti (200 nm) bottom electrode. The Ti electrode was covered with a magnetron-sputtered ITO (10 nm) interlayer that provides a surface capable of binding the phosphonic acid groups of 2PACz to create a hole-selective contact between the perovskite absorber and the Ti electrode. The 2PACz monolayer (∼1 nm) forms a conformal charge-selective dipole layer on the ITO interlayer with negligible resistive losses.^31^ A triple-cation perovskite (Cs_0.05_(MA_0.17_FA_0.83_)_0.95_Pb(I_0.83_Br_0.17_)_3_, where MA is methylammonium and FA is formamidinium) (520 nm), was deposited from a precursor solution in a 4:1 (v/v) mixture of DMF and DMSO using ethyl acetate as the antisolvent to induce crystallization, followed by thermal annealing.^36^ Thermally evaporated C_60_ (20 nm) served as the ETL. To enable top illumination of the substrate-configuration cells, a transparent ITO/MgF_2_ top contact was used. ITO (180 nm) was deposited by magnetron sputtering and MgF_2_ (90 nm) by thermal evaporation. To protect the perovskite and C_60_ layers during magnetron sputtering, a SnO_2_ (45 nm) layer was deposited on top of C_60_ via ALD. The yield of working cells suffered from a suboptimal surface wetting of the perovskite precursor solution on the HTL. This also resulted in variations in *J–V* characteristics and thus in the PCEs. An extra UV-ozone surface treatment of the ITO interlayer before applying the 2PACz HTL improved the quality of the perovskite film. Cells that were not affected by suboptimal film formation performed well. For comparison, a conventional superstrate cell on glass covered with ITO (170 nm) was made in a similar stack design but employing a thermally evaporated opaque top contact consisting of BCP (8 nm) and Ag (100 nm) (Figure 1, cell C). Scanning electron microscopy (SEM) images of the perovskite films deposited for the three configurations A, B, and C shown in Figure 1 do not reveal any significant differences (Figure S1, Supporting Information) and indicate that the perovskite layers formed on ITO/2PACz do not strongly depend on the choice of the substrate underneath [glass/ITO/Ti (A), steel/PAI/Ti (B), or glass (C)].

**Figure 2:**
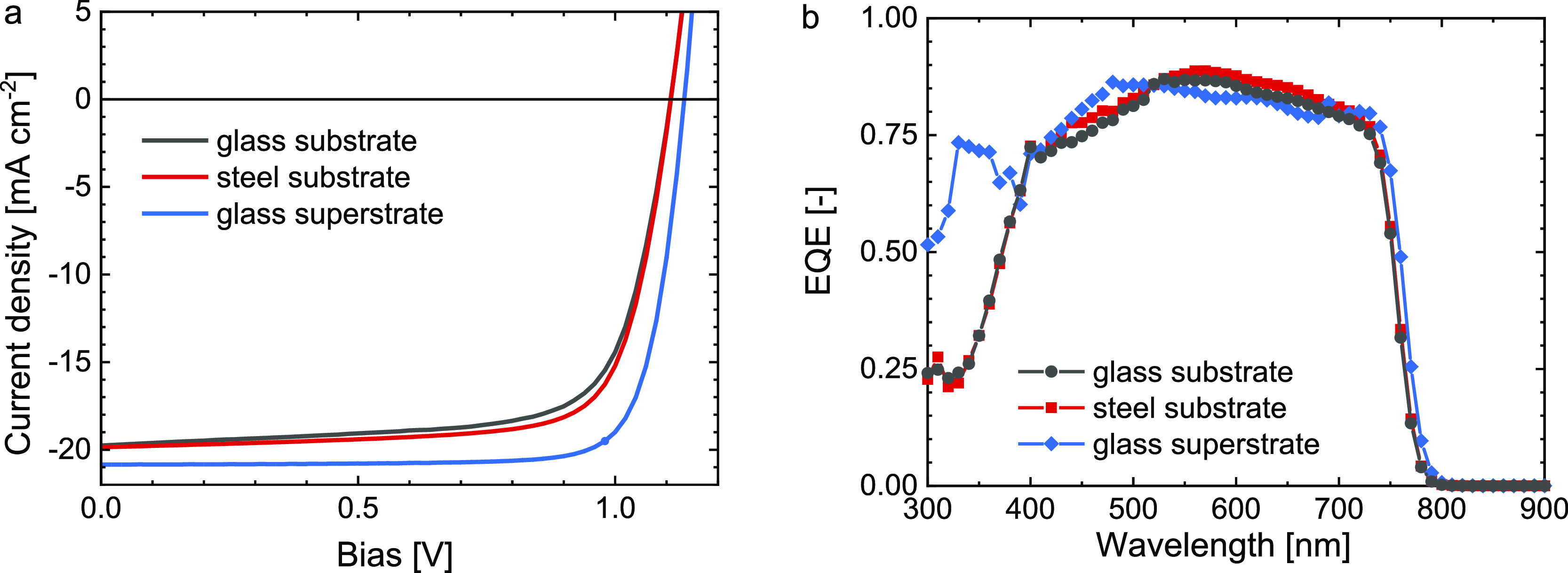
(a) Stabilized *J–V* characteristics of triple-cation perovskite substrate-configuration (on glass and steel) and superstrate-configuration (on glass) solar cells illuminated with simulated AM1.5G light (100 mW cm^–2^). (b) EQE spectra of the same devices recorded with 1 sun equivalent bias light.

The substrate cell on glass (cell A) reaches 15.8% PCE with an open-circuit voltage (*V*_oc_) of 1.11 V, a short-circuit current density (*J*_sc_) of 19.8 mA cm^–2^, and a fill factor (FF) of 0.72 (Table 1 and Figure 2a). Very similar characteristics are found for the substrate cell fabricated on the planarized steel substrate (cell B), with PCE = 16.5% and virtually identical *V*_oc_ = 1.11 V and *J*_sc_ = 19.9 mA cm^–2^, but a slightly higher FF = 0.75. This demonstrates that substrate-configuration cells fabricated on the Ni-plated steel substrate can reach similar performance levels as those made on glass. The conventional p–i–n superstrate cell on glass/ITO (cell C), however, provides a significantly higher PCE of 18.4%, mainly because of an improved *J*_sc_ (20.8 mA cm^–2^) and higher FF (0.81). Device statistics are shown in Figure S2 (Supporting Information).

**Table 1:** Photovoltaic Parameters of Substrate and Superstrate Cells

cell	*J*_sc_ [mA cm^–2^]	*J*_sc,EQE_^a^ [mA cm^–2^]	*V*_oc_ [V]	FF [−]	PCE [%]	PCE_EQE_^a^ [%]
substrate on glass	19.8	19.3	1.11	0.72	15.8	15.4
substrate on steel	19.9	19.7	1.11	0.75	16.5	16.4
superstrate on glass	20.8	20.1	1.13	0.81	19.1	18.4

a Based on integration of the EQE spectrum with the AM1.5G spectrum.

In the visible and near-infrared spectral range, the substrate cells on glass and steel reach a similar EQE to the superstrate cell (Figure 2b). The latter exhibits an improved response in the UV range because of the higher transparency of the bottom-ITO than the top-ITO layer and because absorption of light by the C_60_ layer in the substrate-configuration devices. Integration of the EQE spectra with the AM1.5G spectrum provides refined estimates for the short-circuit current density (*J*_sc,EQE_) and efficiency (PCE_EQE_) that are within a small margin from the values obtained from the *J–V* characteristics (Table 1).

A noticeable difference between the two configurations is the higher FF for the superstrate cell (0.81) than for the substrate cells (0.72–0.75). The difference is, at least in part, caused by the lower sheet resistance of the ITO bottom electrode (∼16 Ω sq^–1^) in the superstrate-configuration cell compared to the ITO top electrode (∼45 Ω sq^–1^) in the substrate-configuration cells. The principal reason for this difference is that thermal annealing at 350–550 °C in air, as commonly used to increase the conductivity of sputtered ITO on glass,^37,38^ is not compatible with the perovskite absorber and the organic charge transport layers.

**Figure 3:**
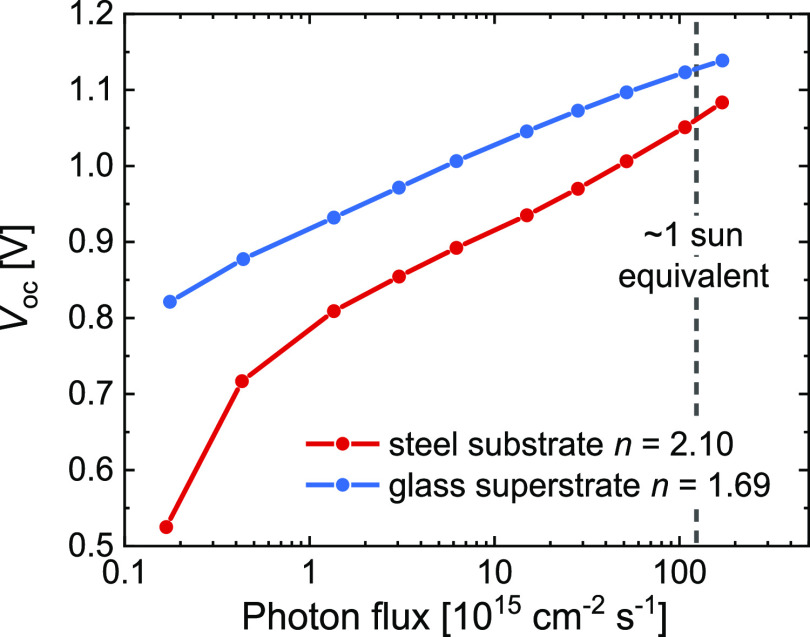
Light-intensity dependence of *V*_oc_ recorded for 530 nm light.

The *V*_oc_ of the substrate cells is slightly lower than for the superstrate cell (1.11 vs 1.13 V). Figure 3 shows the *V*_oc_ as a function of photon flux for both cells in a semilogarithmic plot. The ideality factor *n* = 2.10 for the substrate cell is higher than *n* = 1.69 for the superstrate cell and suggests that charge recombination dynamics at open circuit are dominated by trap-assisted recombination. The steep decrease of *V*_oc_ observed for the substrate cell at the lowest photon flux (<10^15^ cm^–2^ s^–1^) is ascribed to leakage current, originating from shunts between the top and bottom electrodes via pinholes in the active layer. Both the suboptimal wetting of the precursor solution and rough Ti/ITO bottom electrode compared to a smooth glass/ITO electrode contribute this difference.

**Figure 4:**
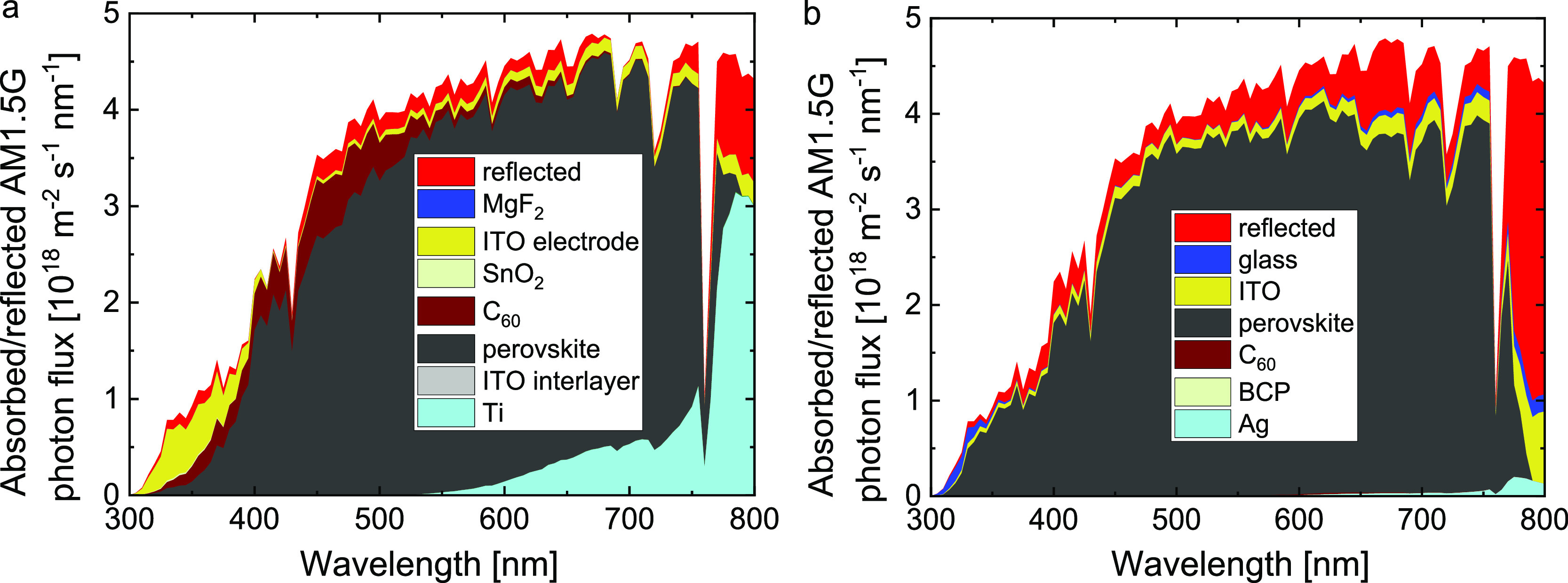
(a) AM1.5G photon flux reflected or absorbed by each individual layer in the substrate cells A and B. (b) Same for the superstrate cell C.

To better understand the difference in *J*_sc_ between the substrate and superstrate cells we modeled the distribution of light of the device stack using the transfer-matrix formalism, using the thickness and the wavelength-dependent refractive index and extinction coefficient of all layers as input parameters (Figures S3 and S4, Supporting Information). In the modeling, we omitted the ∼1 nm 2PACz monolayer because it has a negligible effect. Figure 4 illustrates the absorption by the perovskite layer and the parasitic optical losses of the ancillary layers in the substrate and superstrate cells as the product of the absorptance (or reflectance) determined from optical simulations and the AM1.5G photon flux Φ [cm^–2^ s^–1^ nm^–1^] as a function of wavelength.

The contributions to the photocurrent and the losses can be expressed in current densities by taking as the product of reflected or absorbed photon flux (Φ) with the elementary charge *q* and integrating over a fixed wavelength regime. The current losses as a consequence of optical losses of the substrate-configuration cell add up to 4.4 mA cm^–2^, which is more than the 3.4 mA cm^–2^ for the superstrate reference cell (Table 2). The main losses in the substrate-configuration cell originate from the reflection of photons (0.9 mA cm^–2^) and parasitic absorption by the ITO top electrode (1.0 mA cm^–2^), the C_60_ layer (1.3 mA cm^–2^), and the Ti bottom electrode (1.2 mA cm^–2^). The main optical losses of the superstrate-configuration cell are due to reflection (2.3 mA cm^–2^) and absorption by the ITO bottom electrode (0.8 mA cm^–2^). We note that the reflection loss of the superstrate cell can be reduced to 1.6 mA cm^–2^ when depositing a ∼100 nm antireflective MgF_2_ coating on the glass substrate. The resulting total optical loss in that case would be reduced to 2.8 mA cm^–2^.

**Table 2:** AM1.5G Equivalent Current Absorbed or Reflected in Substrate and Superstrate Cells

substrate cells A and B			superstrate cell C		
layer	thickness [nm]	*q*Φ^a^ [mA cm^–2^]	Layer	thickness [nm]	*q*Φ^a^ [mA cm^–2^]
reflected		0.9	reflected^b^		2.3
MgF_2_	90	∼0	Glass	750 μm	0.2
ITO electrode	180	1.0	ITO electrode	180	0.8
SnO_2_	45	∼0			
C_60_	20	1.3			
perovskite	520	20.4	perovskite	520	21.7
ITO interlayer	10	∼0	C_60_	20	∼0
			BCP	8	∼0
Ti electrode	200	1.2	Ag electrode	100	0.1

a Integration is from 300 to 755 nm, for all layers except for the perovskite layer where integration was up to 800 nm.

b For the superstrate cell, the refection loss can be reduced to 1.6 mA cm^–2^ when using a MgF_2_ (100 nm) antireflective coating.

The optically modeled maximum photocurrent for the substrate cell is 20.4 mA cm^–2^, compared to 21.7 mA cm^–2^ for the superstrate cell (Table 2). The AM1.5G-averaged internal quantum efficiency (IQE) determined from the ratio of *J*_sc,EQE_ (Table 1) and the optically modeled maximum photocurrent is ∼93% for the substrate cell and ∼95% for the superstrate cell. The minor difference between the two IQE values is well within the expected accuracy of the experimental and modeling procedures. The modeling results indicate that the photocurrent of substrate-configuration PSCs can be further enhanced by three different strategies. The first would be to reduce the thickness of the C_60_ layer to a minimum of ∼10 nm, which will reduce the parasitic absorption of photons in the ETL to 0.6 mA cm^–2^ or replace the C_60_ layer with a less absorbing ETL. A second strategy involves replacing the Ti bottom electrode with a more reflective metal such as Cu. This would reduce the absorption by the bottom electrode to ∼0.1 mA cm^–2^. Finally, the absorption of light in the active layer can be increased with a thicker perovskite layer. By combining the three changes, the optical simulations predict a maximum photocurrent of 22.4 mA cm^–2^ for a 650-nm-thick perovskite layer, with a total optical loss of only 2.7 mA cm^–2^. By reducing the optical losses of the substrate-configuration cell, it can reach a similar photocurrent to the superstrate-configuration cell, even when the latter has antireflective coating.

It is of interest to compare the PCE of 16.5% for the p–i–n substrate-configuration cell to the PCE of 14.9% recently reported for n–i–p substrate-configuration cells on the same quality planarized steel.^19^ The n–i–p substrate cells use identical Ti/ITO bottom and ITO/MgF_2_ top electrodes but differ in the charge-selective contact layers. A nanoparticle SnO_2_ layer covered with a PCBA ([6,6]-phenyl-C_61_-butyric acid) monolayer serves as the ETL, while the HTL is composed of a thin thermally evaporated layer of TCTA (tris(4-carbazoyl-9-ylphenyl)amine) (10 nm) covered with MoO_3_ (15 nm). The TCTA/MoO_3_ HTL causes less parasitic absorption and provides marginally higher *J*_sc_ (20.2 vs 19.9 mA cm^–2^). Also, the *V*_oc_ is somewhat higher (1.15 V vs 1.11 V), possibly because the SnO_2_/PCBA layer provides a better energetic alignment with the perovskite than C_60_. The main difference is in the FF which is significantly lower for the n–i–p cell (FF = 0.64 vs 0.75). When comparing the FFs for n–i–p and p–i–n substrate cells on smooth glass instead of rough steel, the difference is much less (0.70 vs 0.72). This suggests that the origin for the difference in FF on steel is not primarily related to different HTLs and ETLs but rather due to a different sensitivity to surface roughness. This seems to be higher for the n–i–p cell than for the p–i–n cell. In accordance, the FF of the n–i–p cells is further reduced to 0.60 when increasing the roughness of the steel substrate,^12^ while there is no loss in FF for the p–i–n cell when going from smooth glass to rougher steel (Table 1). The reason for the difference in sensitivity of the FF to roughness is possibly related to less conformal coverage of the aqueous nanoparticle SnO_2_ dispersion on the corrugated Ti/ITO surface than the 2PACz SAM.

## 4 Conclusions

In summary, an efficient p–i–n PSC has been fabricated on a steel substrate coated with a polymer planarization layer in combination with a transparent top electrode. The substrate-configuration cell was fabricated on a Ti bottom electrode covered with a thin ITO interlayer to enable binding of 2PACz as a self-assembled hole-selective monolayer. Although the processing of these cells must be improved to increase the yield of efficient devices, the best cell made on a polymer-coated Ni-plated battery steel substrate achieved a PCE of 16.5%. Compared to the corresponding superstrate p–i–n cell on glass with a PCE of 18.4%, the main loss is in the FF (0.75 compared to 0.81) due to the high sheet resistance of the ITO top electrode. Optical simulations reveal that the total optical loss of the substrate cell (4.4 mA cm^–2^) is only slightly higher than that of a standard superstrate p–i–n reference cell (3.3 mA cm^–2^). The difference is mainly due to increased parasitic absorption by the C_60_ ETL and the Ti bottom electrode. Strategies to further increase the PCE of substrate-configuration cells can therefore focus on reducing the optical losses in the ETL, enhancing the reflection of the bottom electrode, and using a less resistive transparent top contact.

## Supporting Information Available

SEM images, device statistics (box plots of photovoltaic parameters), wavelength-dependent refractive indices, and extinction coefficients of the layers as used in the optical simulations (PDF)
