# Supplementary material for: p–i–n Perovskite Solar Cells on Steel Substrates
Source: ACS Appl Energy Mater. 2022 Jun 14;5(6):6709–15. doi: 10.1021/acsaem.2c00291 (PMC9241001; doi:10.1021/acsaem.2c00291)
Supplement: Supplementary file 1 — ae2c00291_si_001.pdf [file ae2c00291_si_001.pdf]

Supporting Information

p−i−n Perovskite Solar Cells on Steel Substrates

Benjamin T. Feleki,[1] Ricardo K. M. Bouwer,[2] Valerio Zardetto,[3] Martijn M. Wienk,[1] René A. J. Janssen[1,4]*

[1] Molecular Materials and Nanosystems and Institute for Complex Molecular Systems, Eindhoven University of Technology, P.O. Box 513, 5600 MB Eindhoven, The Netherlands, E-mail: r.a.j.janssen@tue.nl.

[2] Tata Steel, Research and Development, Surface Engineering – Coating Development, IJmuiden, The Netherlands.

[3] TNO, partner in Solliance, High Tech Campus 21, 5656 AE Eindhoven, The Netherlands.

[4] Dutch Institute for Fundamental Energy Research, De Zaale 20, 5612 AJ Eindhoven, The Netherlands.

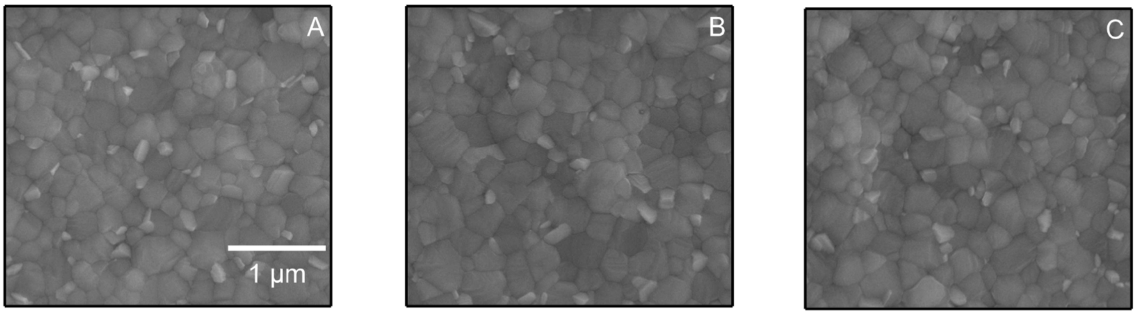

Figure S1. SEM images of triple-cation perovskite $Cs_{0.05}(MA_{0.17}FA_{0.83})_{0.95}Pb(I_{0.83}Br_{0.17})_3$ films. (A) on glass/ITO/Ti/ITO/2PACz. (B) on steel/PAI/Ti/ITO/2PACz. (C) on glass/ITO/2PACz.

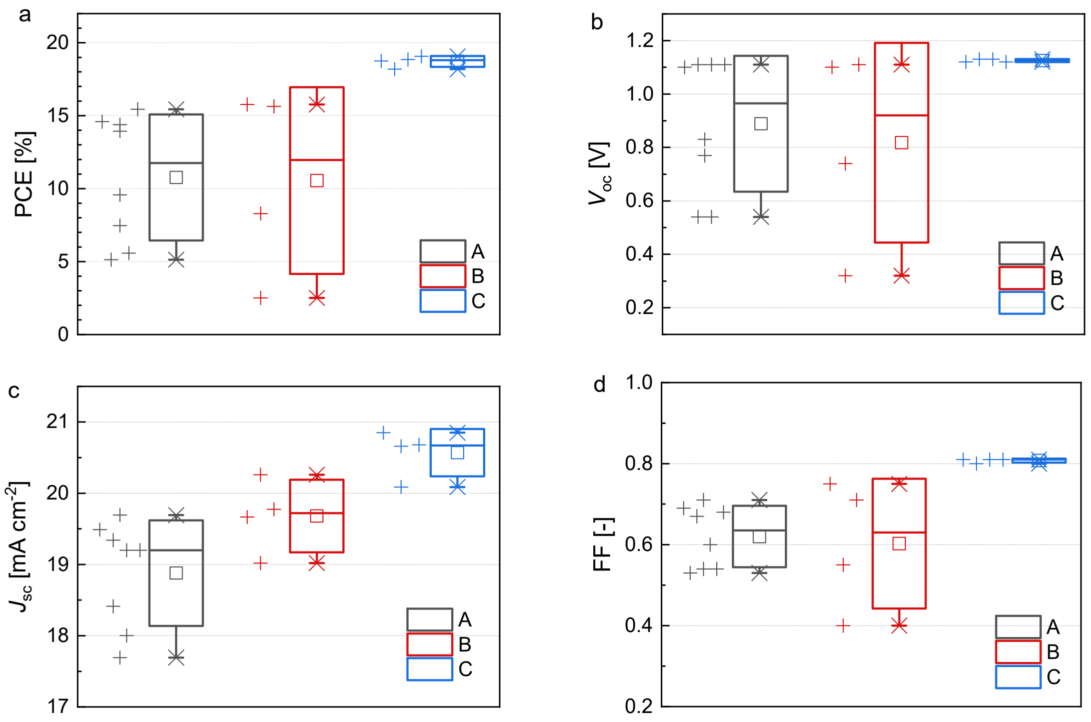

Figure S2. Statistics of fast-scan photovoltaic characteristics of substrate-configuration PCSs on glass (cell A) and steel (cell B), and of the superstrate PSCs on glass (cell C) recorded under illumination with simulated AM1.5G light (100 mW cm^{-2}).

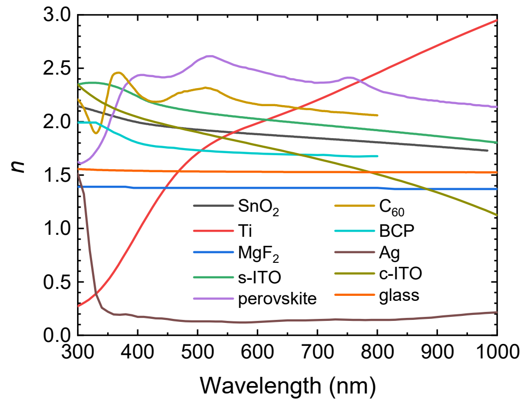

Figure S3. Wavelength dependent refractive indices (n) for the layers used in the optical simulations. s-ITO is sputtered ITO, c-ITO is commercial ITO.

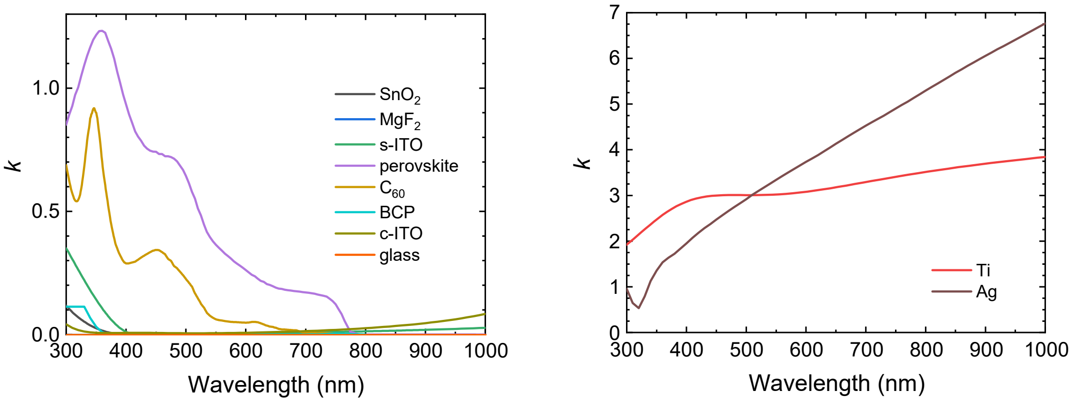

Figure S4. (a) Wavelength dependent extinction coefficients (*k*) for the layers used in the optical simulations. s-ITO is sputtered ITO, c-ITO is commercial ITO.
